# Clinical trial landscape for stem cells in autoimmune and inflammatory diseases: a comprehensive analysis and path forward

**DOI:** 10.1097/MS9.0000000000004148

**Published:** 2025-10-22

**Authors:** Di Wu, Feng Gao, MingJuan Dai, Ning Gao

**Affiliations:** a Department of Hematology, People’s Hospital of Wenshan Prefecture, The Affiliated Hospital of Kunming University of Science and Technology, Wenshan, China; b Department of Neurosurgery, People’s Hospital of Wenshan Prefecture, The Affiliated Hospital of Kunming University of Science and Technology, Wenshan, China; c Department of Nephrology, People’s Hospital of Wenshan Prefecture, The Affiliated Hospital of Kunming University of Science and Technology, Wenshan, China; d Department of Stomatology, People’s Hospital of Wenshan Prefecture, The Affiliated Hospital of Kunming University of Science and Technology, Wenshan, China

**Keywords:** autoimmune diseases, clinical trials, mesenchymal stem cells, regenerative medicine, stem cell therapy

## Abstract

**Background:**

Autoimmune and inflammatory diseases present significant therapeutic challenges due to their chronic nature and limited response to conventional treatments. Stem cell-based therapies have emerged as a promising approach, leveraging their immunomodulatory and regenerative properties to address treatment-resistant cases.

**Methods:**

We conducted a comprehensive analysis of 1136 stem cell clinical trials from the TrialTrove database (October 2024), focusing on therapeutic efficacy, trial progression patterns, and geographic distribution. Key clinical outcomes and safety profiles were systematically evaluated. A choropleth map (Figure 1C) summarizes national activity across continents, with darker shading indicating more trials.

**Results:**

Stem cell trials showed substantial growth over two decades, increasing from 11 trials annually (2000-2004) to a peak of 73 trials in 2019. However, translation to late-phase studies remains limited, with only 10.2% of trials advancing to Phase III. Notable therapeutic successes include a 92.5% survival rate in systemic lupus erythematosus using umbilical cord mesenchymal stem cells and a 56.3% remission rate in Crohn’s disease with adipose-derived mesenchymal stem cells versus 38.6% with placebo (*P* = 0.010). Safety analyses across 36 randomized controlled trials involving 2076 participants demonstrated favorable safety profiles. These dynamics are visualized in Figure 1A, using stacked bars by phase with a superimposed total line to highlight the 2019 inflection. The distribution by phase together with the current recruitment status is detailed in Figure 1B.

**Conclusions:**

Stem cell-based therapies demonstrate significant therapeutic potential for autoimmune and inflammatory diseases. To accelerate clinical translation, the field must address standardization challenges, enhance late-phase trial development, and establish robust regulatory frameworks. These efforts are essential for realizing the full therapeutic potential of stem cell therapies.

*To the Editor,*

Autoimmune and inflammatory diseases, including systemic lupus erythematosus (SLE), Crohn’s disease, rheumatoid arthritis (RA), and multiple sclerosis (MS), continue to pose significant challenges in clinical practice. These conditions are characterized by chronic inflammation, progressive tissue damage, and substantial impacts on patient quality of life[1]. While advances in immunosuppressive therapies and biologics have improved outcomes, many patients experience incomplete responses, treatment failures, or significant adverse effects[2].

This review adheres to the 2025 TITAN Guidelines governing the declaration and use of AI in scholarly communication[3].

This therapeutic gap has driven research into stem cell-based approaches, which offer potential advantages through their ability to modulate immune responses while promoting tissue regeneration[4]. Unlike conventional immunosuppressive strategies, stem cells may potentially restore immune tolerance and address underlying pathophysiology of autoimmune diseases[5].

## Clinical Trial landscape: growth and development challenges

**Figure 1.**
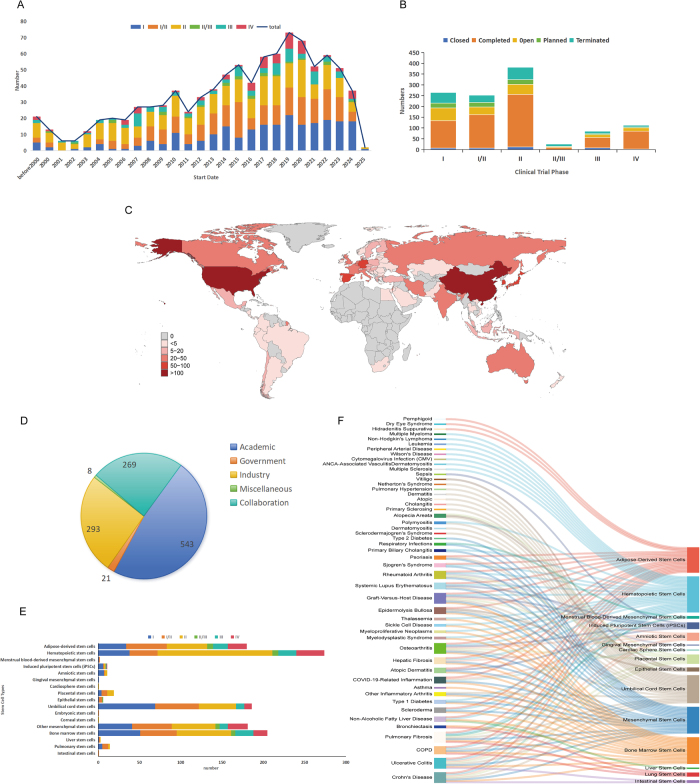
Global clinical trial landscape of stem cell therapies for autoimmune and inflammatory diseases (TrialTrove, cut-off October 2024; N = 1136). (A) Annual number of trials by phase (I, I/II, II, II/III, III, IV) from 2000 to 2024, with total shown as a line. (B) Trial status by phase (Closed, Completed, Open, Planned, Terminated). (C) Geographic distribution of trials by country (choropleth; darker shading indicates more trials). (D) Sponsorship categories (Academic, Industry, Collaboration, Government, Miscellaneous). Two trials with undisclosed sponsors are not displayed. (E) Indication-level trial counts stratified by phase for major autoimmune and inflammatory diseases. (F) Sankey diagram linking diseases to stem cell sources and trial phases. Abbreviations: MSC, mesenchymal stem cell; UC-MSC, umbilical cord MSC; AD-MSC, adipose-derived MSC.

Our systematic analysis of 1136 stem cell trials reveals substantial growth in research activity over the past two decades. Early trial activity was modest, averaging 11 new studies annually between 2000 and 2004, increasing to 24 annual trials during 2005-2009, followed by acceleration from 2010 onward, reaching a peak of 73 trials in 2019. These dynamics are visualized in Figure 1A, using stacked bars by phase with a superimposed total line to highlight the 2019 inflection.

However, significant challenges persist in advancing trials beyond early-phase studies. Phase II trials account for 33.0% of all studies, while only 10.2% have progressed to Phase III confirmatory trials, and merely 3.9% have reached Phase IV. This pattern indicates substantial hurdles in translating early-phase results into large-scale studies necessary for regulatory approval. The distribution by phase together with the current recruitment status is detailed in Figure 1B.

Geographically, North America and Asia lead in trial activity, with the United States and China conducting the most research. Europe also contributes significantly, with notable activity in Spain, Germany, and Italy. Supportive regulatory frameworks, such as China’s 2015 “Administrative Measures on Stem Cell Clinical Research,” have accelerated trial initiation[4]. A choropleth map (Figure 1C) summarizes national activity across continents, with darker shading indicating more trials.

Sponsorship patterns reveal that academic institutions initiate nearly half (48.0%) of all trials, followed by industry sponsors (25.9%) and collaborative initiatives (23.8%). Limited government involvement represents a potential opportunity for increased public investment. These sponsorship patterns are summarized in Figure 1D.

To orient the disease-specific synthesis that follows, Figure 1E–F summarizes indication-level trial counts by phase and links diseases with stem-cell sources.

## Clinical evidence: progress and limitations

Systemic Lupus Erythematosus: Umbilical cord mesenchymal stem cells (UC-MSCs) have shown potential in treating refractory SLE. A multicenter Phase II trial (identifier anonymized) involving patients with severe, treatment-resistant SLE reported a 92.5% overall survival rate[5]. While the study documented improvements in disease activity indices, including reductions in SLEDAI scores (p < 0.001), these results require validation in larger, controlled studies. Notably, no transplantation-related adverse events were reported, though long-term safety data remain limited.

Crohn’s Disease: Adipose-derived mesenchymal stem cells have achieved the most advanced regulatory progress in inflammatory bowel disease. The ADMIRE-CD trial demonstrated that darvadstrocel (Cx601) achieved a combined remission rate of 56.3% at 52 weeks compared with 38.6% in the placebo group (*P* = 0.010)^[6,7]^. While this led to FDA breakthrough therapy designation, questions remain regarding optimal patient selection and long-term durability of responses.

Inflammatory Arthritis: A meta-analysis of 36 randomized controlled trials involving 2076 participants showed improvements in pain reduction and functional outcomes across various metrics[8]. In osteoarthritis, mesenchymal stem cells from different sources demonstrated reductions in Visual Analog Scale (VAS) pain scores and improvements in the Western Ontario and McMaster Universities Arthritis Index (WOMAC). However, heterogeneity in study designs and outcome measures limits definitive conclusions about optimal treatment protocols.

Type 1 Diabetes: Small-scale studies using reprogrammed pluripotent stem cells to generate insulin-secreting islet cells have demonstrated temporary insulin independence in some patients following transplantation[9]. However, these preliminary results require larger studies with longer follow-up to assess durability and safety.

## Persistent challenges and limitations

Despite encouraging preliminary results, several critical barriers limit widespread clinical translation. Manufacturing variability remains a significant challenge, with differences in cell preparation protocols, donor selection criteria, and expansion methods affecting therapeutic consistency across studies. This variability complicates result interpretation and hinders standardized treatment protocol development.

Standardization challenges extend to dosing regimens, delivery methods, and potency assays, making it difficult to design robust, reproducible trials. While stem cell therapies generally demonstrate acceptable safety profiles in short-term studies, questions remain regarding long-term safety and durability of therapeutic effects, particularly for allogeneic cell products that may carry immunogenic risks.

Regulatory challenges impede progress, with fragmentation across countries limiting cross-border collaboration and slowing commercialization. High manufacturing costs, complex logistical requirements, and quality control challenges further limit accessibility and scalability. Additionally, many trials lack adequate control groups or blinding, limiting the strength of evidence.

## Strategic priorities for field advancement

To address these limitations and accelerate clinical translation, several priorities emerge: (1) development of standardized manufacturing protocols and validated biomarkers for therapeutic potency; (2) advancement of more trials into late-phase development to address the current Phase II bottleneck; (3) integration of emerging technologies including gene-editing and AI-driven patient stratification^[10,11]^; and (4) international coordination to address regulatory fragmentation and promote data sharing.

Enhanced trial design with appropriate controls, longer follow-up periods, and standardized outcome measures will be essential for generating robust evidence. Cost-effectiveness analyses and real-world evidence generation will also be crucial for health care system adoption.

## Future perspectives and conclusion

The stem cell therapy field shows promise but faces significant translational challenges. While early results suggest potential benefits in certain autoimmune and inflammatory conditions, the field requires continued rigorous investigation to establish safety, efficacy, and cost-effectiveness.

The limited success of darvadstrocel in Crohn’s disease provides a framework for regulatory approval, though broader application requires addressing manufacturing standardization, regulatory harmonization, and late-phase trial development challenges.

Stem cell-based therapies represent a potential therapeutic option for autoimmune and inflammatory diseases, offering novel mechanisms that may address disease pathophysiology. However, the clinical evidence from over 1100 trials reveals both promise and significant challenges in translation to widespread clinical practice.

Moving forward, collaboration between academic researchers, industry sponsors, regulatory agencies, and patient advocacy groups will be essential. Patients with limited treatment options may benefit from these innovative approaches, but only through rigorous scientific development with careful attention to safety, efficacy, and cost-effectiveness standards.

The evidence supports cautious investigation rather than immediate clinical optimism. With sustained commitment to addressing current challenges through rigorous research, these therapies may eventually contribute to improved treatment paradigms for patients with autoimmune diseases.

## Data Availability

The datasets generated and/or analyzed during the current study are available from the corresponding author on reasonable request.

## Research Registration Unique Identifying Number (UIN)

Not applicable, as this study did not involve human participants.

## Peer and provenance statement

This article was not commissioned and was externally peer reviewed.
